# Public Preferences for Determining Eligibility for Screening in Risk-Stratified Cancer Screening Programs: A Discrete Choice Experiment

**DOI:** 10.1177/0272989X231155790

**Published:** 2023-02-14

**Authors:** Rebecca A. Dennison, Lily C. Taylor, Stephen Morris, Rachel A. Boscott, Hannah Harrison, Sowmiya A. Moorthie, Sabrina H. Rossi, Grant D. Stewart, Juliet A. Usher-Smith

**Affiliations:** Primary Care Unit, Department of Public Health and Primary Care, University of Cambridge, Cambridge, UK; Primary Care Unit, Department of Public Health and Primary Care, University of Cambridge, Cambridge, UK; Primary Care Unit, Department of Public Health and Primary Care, University of Cambridge, Cambridge, UK; School of Clinical Medicine, University of Cambridge, Cambridge, UK; Centre for Cancer Genetic Epidemiology, Department of Public Health and Primary Care, University of Cambridge, Cambridge, UK; PHG Foundation, University of Cambridge, Cambridge, UK; Department of Surgery, University of Cambridge, Cambridge, UK; Department of Surgery, University of Cambridge, Cambridge, UK; Primary Care Unit, Department of Public Health and Primary Care, University of Cambridge, Cambridge, UK

**Keywords:** discrete choice experiment, population survey, public acceptability, cancer screening, health policy, risk factors

## Abstract

**Background:**

Risk stratification has been proposed to improve the efficiency of population-level cancer screening. We aimed to describe and quantify the relative importance of different attributes of potential screening programs among the public, focusing on stratifying eligibility.

**Methods:**

We conducted a discrete choice experiment in which respondents selected between 2 hypothetical screening programs in a series of 9 questions. We presented the risk factors used to determine eligibility (age, sex, or lifestyle or genetic risk scores) and anticipated outcomes based on eligibility criteria with different sensitivity and specificity levels. We performed conditional logit regression models and used the results to estimate preferences for different approaches. We also analyzed free-text comments on respondents’ views on the programs.

**Results:**

A total of 1,172 respondents completed the survey. Sensitivity was the most important attribute (7 and 11 times more important than specificity and risk factors, respectively). Eligibility criteria based on age and sex or genetics were preferred over age alone and lifestyle risk scores. Phenotypic and polygenic risk prediction models would be more acceptable than screening everyone aged 55 to 70 y if they had high discrimination (area under the receiver-operating characteristic curve ≥0.75 and 0.80, respectively).

**Limitations:**

Although our sample was representative with respect to age, sex, and ethnicity, it may not be representative of the UK population regarding other important characteristics. Also, some respondents may have not understood all the information provided to inform decision making.

**Conclusions:**

The public prioritized lives saved from cancer over reductions in numbers screened or experiencing unnecessary follow-up. Incorporating personal-level risk factors into screening eligibility criteria is acceptable to the public if it increases sensitivity; therefore, maximizing sensitivity in model development and communication could increase uptake.

**Highlights:**

- The public prioritized lives saved when considering changing from age-based eligibility criteria to risk-stratified cancer screening over reductions in numbers of people being screened or experiencing unnecessary follow-up.
- The risk stratification strategy used to do this was the least important component, although age plus sex or genetics were relatively preferable to using age alone and lifestyle risk scores.
- Communication strategies that emphasize improvements in the numbers of cancers detected or not missed across the population are more likely to be salient than reductions in unnecessary investigations or follow-up among some groups.
- Future research should focus on developing implementation strategies that maximize gains in sensitivity within the context of resource constraints and how to present attributes relating to specificity to facilitate understanding and informed decision making.

## Graphical abstract

**Graphical Abstract:**
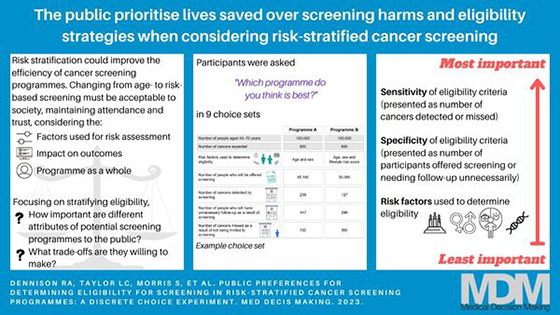
This is a visual representation of the abstract.

## Introduction

Population-level cancer screening programs aim to save lives and reduce health care and societal burden of specific cancers through prevention or by detection at an early stage when treatment is more effective.^1^ Despite considerable benefits, there are harms associated with screening. These include diverting resources away from other uses within health care systems, overdiagnosis, false-positive results, and psychological harms such as anxiety and false reassurance.^1^ Most current programs screen based on age, sometimes restricted to one sex; for example, everyone aged 50 to 74 y is invited for bowel cancer screening, and all women aged 50 to 69 y are invited for breast cancer screening in many European countries.^2^

However, factors other than age also influence individual cancer risk. In the United Kingdom in 2015, 15% of cancer cases were attributable to tobacco smoking, 6% to overweight and obesity, and 3% to 4% each to radiation, occupation, infections, alcohol, and insufficient fiber.^3^ Among all cancer cases, 5% to 10% are anticipated to be due to genetic predisposition.^4^ These risk factors are not distributed equally across the population, with the result that individuals of the same age can have very different risks of particular cancers. For example, people with healthy lifestyles or no family history have up to half the risk of bowel cancer compared with those with the least favorable lifestyles or affected first-degree relatives.^5,6^ Accordingly, the average net benefit of screening is greater among subgroups of the population at higher cancer risk than subgroups at lower risk, leading to calls for more personalized or stratified screening.^7,8^

Numerous risk prediction models enable estimation of an individual’s personal risk of specific cancers.^9–13^ A key challenge in moving to risk-stratified screening, however, is identifying the best strategy for implementation. Cancer screening programs as a whole, including the eligibility criteria, must be acceptable to the population.^14^ Our recent qualitative research found that informed members of the public supported implementing risk stratification to determine eligibility.^15^ While some participants thought that models with the best predictive performance should be used, others felt that only nonmodifiable risk factors should be included.^15^ Furthermore, the public are keen to discover their genetic risk of cancer and for this to be used for risk calculations and stratification.^16,17^ However, for genetics to inform risk-based breast screening, how genetic testing is accessed was found to be more important than sensitivity and specificity of the risk assessment.^18^ Although test sensitivity and mortality reduction are frequently the most important attributes of cancer screening programs to the public in general,^19^ studies on risk stratification have focused on the risk factors themselves and not the potential impact on screening outcomes. No studies to our knowledge have explored the importance of such attributes relating to eligibility criteria, specifically, how accurate risk prediction models need to be to be acceptable when used to inform screening eligibility.

Discrete choice experiments (DCEs) quantify strength of preference by asking respondents to select between hypothetical strategies with different characteristics (attributes) in a series of questions.^20^ They are, therefore, useful for studying the determinants of choices that cannot be easily observed or are not routinely available. Multiple DCEs studying preferences for cancer screening have been conducted^19^ but not in the context of risk stratification for eligibility.

Therefore, the aim of this study was to describe the relative importance of different attributes of potential approaches to identify eligibility for screening within risk-stratified screening programs and the tradeoffs individuals are willing to make between these. We asked respondents to consider age as the current approach for determining eligibility for screening, and risk stratification using sex, phenotypic/lifestyle risk, or genetic risk in addition to age.

## Methods

Our DCE was designed and is reported in line with published recommendations.^20,21^ Two patient and public involvement (PPI) representatives contributed to its design. Ethical approval was obtained from the University of Cambridge Psychology Research Ethics Committee (PRE.2021.046).

### Participants and Recruitment

Using Prolific (www.prolific.co), an online recruitment platform, we recruited individuals who were residents of the United Kingdom and representative of the population in terms of age, sex, and ethnicity. After seeing a brief summary of the study, potential participants viewed the full participant information sheet online (see the University of Cambridge repository, http://doi.org/10.17863/CAM.86460) and gave online consent. All respondents were free to withdraw for any reason at any time by returning their submissions in Prolific. Respondents who completed the survey were compensated an anticipated £7.50/h by Prolific.

### Survey Design

The survey (Supplementary File 1) had 3 components: 1) demographic questions plus numeracy, perception of cancer risk and worry, and attitudes toward cancer screening using validated questions where available^22–26^ (presented over 8 screens); 2) guidance and the DCE questions (28 screens); and 3) evaluation questions, including ease of competing the DCE and ranking the importance of the attributes (1 screen). The survey was hosted on the Gorilla Experiment Builder (www.gorilla.sc).^27^ It was piloted in online face-to-face interviews (*n* = 4) and electronically (*n* = 8) with members of a PPI panel to identify any areas of misunderstanding, common errors, and technical functionality. Following piloting, we simplified some wording in the participant information leaflet and consent form, slightly revised the order of the survey, and added additional signposting within the survey (explaining what was coming next and the number of questions in the DCE).

Table 1 details the attributes and levels that were included in the DCE. Sensitivity, specificity, and risk factors used to determine eligibility were selected following reviews of cancer screening and DCE literature, our recent community jury study,^15^ and consultation with topic experts and PPI representatives. Levels were assigned based on published literature to reflect plausible and clinically relevant ranges while avoiding extreme values to limit grounding effects. Because they are complex concepts for lay individuals to understand, we translated the sensitivity and specificity of eligibility criteria (underlying attributes) into a number of screening outcomes based on a population of 100,000 people aged 40 to 70 y with 600 cases of cancer (displayed attributes). These relate to both screening eligibility and cancer outcomes. Supplementary Table S1 shows how the displayed attributes were calculated from the number of true positives and negatives and false positives and negatives of the eligibility criteria and a screening test at each level of sensitivity and specificity. We informed respondents that screening test sensitivity and specificity, prevalence of cancer, the impact of detection through screening on survival, and the burden or cost of the screening tests were constant throughout the scenarios.

**Table 1:** Attributes and Levels Included in the Discrete Choice Experiment

Attribute Name	Attribute Description	Levels Available
Underlying attributes (accuracy of eligibility criteria)		
Risk factors used to determine eligibility	Risk factors used to determine eligibility for screening	•Age •Age and sex •Age, sex, and lifestyle risk score •Age and genetic risk score
Sensitivity	Sensitivity of the eligibility criteria	•40% •55% •75% •90%
Specificity	Specificity of the eligibility criteria	•25% •55% •70% •80%
Displayed attributes (screening outcomes)		
Risk factors used to determine eligibility	Risk factors used to determine eligibility for screening	•Age •Age and sex •Age, sex, and lifestyle risk score •Age and genetic risk score
Number of people who will be offered screening	Number of people who will be offered screening out of a population of 100,000 people aged 40 to 70 y (a measure of specificity of eligibility criteria)	•20,270 •30,210 •45,120 •74,950
Number of cancers detected by screening	Number of cancers detected following diagnostic testing after a positive screening test (a measure of sensitivity of eligibility criteria, cancer prevalence, and screening test performance)	•127 •175 •239 •286
Number of people who will have unnecessary follow-up as a result of screening	Number of people who will have diagnostic testing after a positive screening test but not have cancer (a measure of specificity of eligibility criteria, cancer prevalence, and screening test performance)	•199 •298 •447 •746
Number of cancers missed as a result of not being invited to screening	Number of cancers missed as a result of not being identified as at a high-enough risk of cancer to be invited to screening (a measure of sensitivity of eligibility criteria and cancer prevalence)	•60 •150 •270 •360

The second section of the survey began with an explanation of the DCE attributes and levels. For example, we explained that cancer will be detected in some people with a positive screening test and many “will then be offered treatment and their lives could be saved.” We set an example with 3 simple questions to facilitate and check understanding. Each participant was then randomized 1:1:1:1 to a block of 9 questions: 1 of 2 sets of 8 questions, plus 1 rationality check question, presented in 1 of 2 orders. The combination of levels presented across 16 questions was generated using the Stata command “dcreate” to maximize the efficiency of the design, “blockdes” was then used to split the questions into 2, and then 2 orders were randomly assigned. The fifth question in each block was a rationality check, in which 1 of the alternatives was clearly superior based on the researchers’ a priori assumptions. The aim of the rationality check was to identify respondents whose choice behavior violated common rationality axioms.

In each question, exemplified in Supplementary File 1, respondents chose between 2 programs (forced-choice elicitation). There was no opt-out option because we sought to determine tradeoffs between different designs, not whether screening programs should exist. One question was displayed per page, and respondents were not able to review or change their answers, which they were told in advance.

### Statistical Analysis

Survey data were downloaded from Gorilla. We summarized participant characteristics and the evaluation using descriptive statistics. All analyses were performed using Stata 15 (StataCorp, College Station, TX, USA) or Microsoft Excel, and *P* values <0.05 were considered statistically significant throughout.

Preferences for different attributes were analyzed using a fixed-effects conditional logit regression model.^28^ Respondents who failed the rationality check were excluded from the main analysis, and the rationality check itself was not included in the models. We approached these analyses in 2 ways. The first used the underlying attributes (risk factors included and sensitivity and specificity of the eligibility criteria), and the second used the attributes displayed to respondents that were calculated from the underlying attributes (risk factors included and screening outcomes for the population). As the displayed attributes were not independent, this second analysis was separated into two so that only one measure of sensitivity and specificity was included in each model. The results from each analysis were then compared descriptively.

We calculated the relative importance of categorical attributes (risk factors) as the difference in coefficients between the most and least preferred level of each attribute.^29^ Continuous attributes (all other attributes) were measured on different scales; therefore, we calculated the relative importance by multiplying each coefficient by its lowest and highest level before finding the difference.

A second set of 11 models were run, with respondents stratified according to demographic characteristics, numeracy, and views on cancer and screening, which were determined a priori by the study team. Binary subgroups were created around median or meaningful cutoffs. We used chi-square tests to assess differences in the relative value of characteristics among these subgroups.

The results of the conditional logit regression models using the underlying attributes were then used to calculate the probability that different programs would be selected. First, we calculated the probability that a range of programs with high (80%), medium (60%), and low (40%) sensitivity and specificity would be preferred, compared with a program using age to determine eligibility with medium sensitivity and specificity. Second, to account for the fact that current risk prediction models do not have both high sensitivity and high specificity, we calculated the probability that a selection of realistic risk prediction models would be preferred, using screening everyone at age 55 y as the comparator. For this analysis, we calculated the sensitivity and specificity of screening everyone (in a population aged 40–74 y) older than 55 y using the combined age-specific incidence of bowel and lung cancer and demographic statistics for the United Kingdom.^30,31^ Bowel and lung cancer were selected because they are the 2 most common cancers in the United Kingdom that affect both men and women.^30^ We then estimated the corresponding sensitivity of screening the same population using risk prediction models with symmetrical receiver-operating characteristic (ROC) curves with areas under the ROC curve (AUCs) of 0.70 to 0.85, as well as different age and sex cutoffs using UK statistics.^30,31^ Specificity was fixed at 48% (the specificity of screening everyone at 55 y) to keep the number of people screened constant and enable comparison across programs.

In addition, we analyzed any free-text comments given in the evaluation using thematic analysis to understand respondents’ strategies for completing the DCE and their views on individual attributes. Two researchers (R.A.D., a researcher with qualitative expertise, and L.C.T., a PhD student new to qualitative research) familiarized themselves with a random sample of 240 (20%) comments split equally between those who found it easy or difficult to choose between the programs and developed initial codes independently. They then discussed and agreed the coding frame, recoded the initial sample of comments to ensure agreement, and coded half of the remaining comments each. Through discussion with other researchers, key themes around the respondents’ views on the attributes were developed.

### Role of the Funding Source

The funders of the study had no roles in the study design, data collection, data analysis, data interpretation, or writing of the report.

## Results

### Participants

The survey was launched on 6 September 2021 and remained live until the target sample size was achieved (approximately 17 h). At this time, 55,207 Prolific participants met the eligibility criteria for the study. Based on Prolific’s demographic data, approximately 85% of these were White, 68% were female, 86% lived in England, 55% had a university-level education, and 47% considered themselves to be in the top 5 deciles of socioeconomic status.

The participant information leaflet was viewed by 1,212 participants, and 40 participants did not complete the survey (97% completion rate). The DCE was therefore completed by 1,172 respondents (239 respondents in each block). Eighty-eight (7.5%) respondents failed the rationality check and so were excluded from the main analysis. Those who failed the rationality check did not differ from all respondents in terms of demographic characteristics (Table 2). Of the respondents, 52.3% were female and 57.3% were aged 40 to 70 y. Half had a university-level education (51.9%), and 5.8% and 39.0% had a personal or family history of cancer, respectively.

**Table 2:** Participant Characteristics

	All Respondents, *n* (%) (*N* = 1,172)	Passed Rationality Check, *n* (%) (*n* = 1,084)	*P* Value for Difference^a^
Age, y			0.99
<30	256 (21.8)	235 (21.7)	
≥30 and <50	419 (35.8)	383 (35.3)	
≥50 and <70	446 (38.1)	417 (38.5)	
≥70	51 (4.4)	49 (4.5)	
Sex and gender			
Sex (female)	613 (52.3)	564 (52.0)	0.90
Sex (missing)	0	0	
Gender identity same as sex (yes)	1,157 (98.7)	1,069 (98.6)	0.98
Gender identity same as sex (missing)	7 (0.6)	7 (0.6)	
Ethnicity			0.99
Asian or Asian British	88 (7.5)	79 (7.3)	
Black or African or Caribbean or Black	36 (3.1)	32 (3.0)	
White	1,003 (85.6)	934 (86.2)	
Mixed or multiple ethnic group	32 (2.7)	27 (2.5)	
Other	13 (1.1)	12 (1.1)	
Education			1.00
Finished school at or before the age of 15 y	14 (1.2)	12 (1.1)	
Completed GCSEs, O Levels, or equivalent	140 (12.0)	128 (11.8)	
Completed A Levels or equivalent	244 (20.8)	226 (20.8)	
Completed further education but not a degree	165 (14.1)	156 (14.4)	
Completed a bachelor’s degree	407 (34.7)	377 (34.8)	
Completed a master’s degree or PhD	201 (17.2)	184 (17.0)	
Missing	1 (0.1)	1 (0.1)	
Social grade based on chief income earner’s occupation			0.99
ABC1 (middle class)	852 (72.7)	788 (72.7)	
C2DE (working class)	311 (26.5)	287 (26.5)	
Missing	9 (0.8)	9 (0.8)	
Smoking			0.98
Never smoked	666 (56.8)	625 (57.7)	
Used to smoke	394 (33.6)	363 (33.5)	
Smoke up to 20	98 (8.4)	85 (7.8)	
Smoke 20 or more	9 (0.8)	7 (0.6)	
Missing	5 (0.4)	4 (0.4)	
Weight (self-reported)			1.00
Underweight	27 (2.3)	25 (2.3)	
About the right weight	460 (39.3)	418 (38.6)	
Slightly overweight	526 (44.9)	493 (45.5)	
Very overweight	158 (13.5)	147 (13.6)	
Missing	1 (0.1)	1 (0.1)	
History of cancer			
Personal (yes)	68 (5.8)	65 (6.0)	0.76
Personal (missing)	4 (0.3)	2 (0.2)	
Family history (yes)	457 (39.0)	429 (39.6)	0.93
Family history (don’t know or missing)	18 (1.5)	18 (1.7)	
Close friend or partner (yes)	673 (57.4)	619 (57.1)	0.97
Close friend or partner (don’t know or missing)	14 (1.2)	12 (1.1)	

a *P* value based on the χ^2^ test for the difference between all respondents and those who passed the rationality check.

Supplementary Table S2 summarizes respondents’ views on cancer and screening. More than half of all respondents (55.1%) thought they were likely to get cancer in the next 10 years, but 56.5% had rarely or never thought about this over the previous month. Almost all respondents thought that the benefits of cancer screening always (58.0%) or sometimes (39.8%) outweighed the possible negatives.

The respondents took a median 14.5 min to complete the survey (interquartile range 11.0–19.4 min). Most answered at least 2 of the 3 numeracy questions (80.9%) or DCE understanding questions (83.2%) correctly (Supplementary Table S3). Half (50.6%) reported that it was easy to select between programs in the DCE, while the other half (49.4%) found it difficult (Supplementary Table S3 and Figure S1).

### Regression Results

Respondents favored screening programs that determined screening eligibility using age and sex or genetics over age alone or a phenotypic risk score (Table 3 using underlying attributes; Supplementary Table S4 using displayed attributes). Age plus sex or genetics were equally preferable. Respondents also favored programs in which the eligibility criteria better estimated who would develop cancer. Sensitivity (or numbers of cancers detected or missed) was the most important attribute, being approximately 7 times more important to respondents than specificity (or number of participants offered screening or needing follow-up unnecessarily) and 11 to 14 times more important than risk factors.

**Table 3:** Conditional Logit Regression Using Underlying Attributes plus Relative Importance of Attributes

Attribute	Coefficient (95% Confidence Interval)	*P* Value for Coefficient	Relative Importance (%)
Risk factors in model			7.4
Age	Reference		
Age and sex	**0.239 (0.136–0.342)**	**<0.001**	
Age, sex, and lifestyle risk score	−0.051 (−0.168–0.066)	0.39	
Age and genetic risk score	**0.242 (0.152–0.332)**	**<0.001**	
Sensitivity^a^	**0.063 (0.061–0.066)**	**<0.001**	80.9
Specificity^b^	**0.008 (0.007–0.010)**	**<0.001**	11.7

*N* = 1,084; number of observations = 17,344; pseudo *R* ^2^ = 0.3953.

a Represented as the number of cancers detected by screening and the number of cancers missed as a result of not being invited to screening in the questionnaire.

b Represented as the number of people who will be offered screening and the number of people who will have unnecessary follow-up as a result of screening in the questionnaire.Bold text highlights statistically significant results (*p*<0.05).

There were several statistically significant differences in responses between demographic subgroups (Supplementary Table S5), but none changed the relative order of attributes, and sensitivity was consistently dominant. Of note, which risk factors were included was significantly more important for working class respondents than those of middle class and for respondents with a family history of cancer compared with those without. Specificity was comparatively less important for those at lower social grades and non-White ethnicity. Respondents with higher numeracy or better understanding of the DCE had significantly stronger preferences for higher sensitivity and specificity. Compared with respondents who said, “it depends,” those who considered that the benefits of screening always outweigh the harms had smaller preferences for higher specificity. There was also a significant difference between the regression results by ease of selecting between programs: sensitivity was more important to those who found the decision easy.

The relative order of attributes again remained unchanged in the sensitivity analysis (Supplementary Table S6). However, sensitivity was more important to the set of respondents who passed the rationality check than to all respondents, although differences were small in absolute terms (sensitivity coefficient 0.063 [0.061–0.066] versus 0.054 [0.052–0.056]).

### Predicted Probabilities

**Figure 1:**
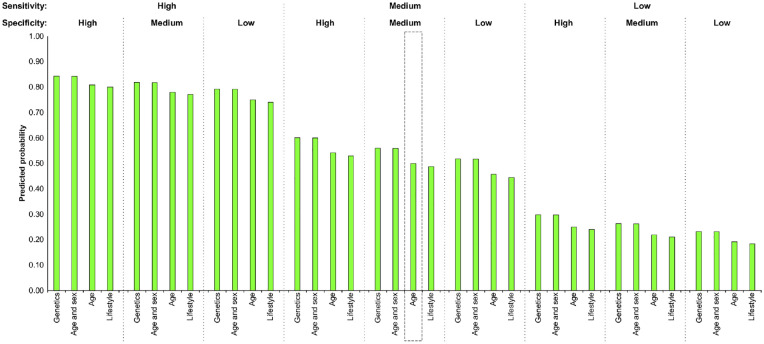
Predicted probabilities of choosing a specified screening program compared to one based on age at medium sensitivity and specificity. Bars indicate the probability that respondents would choose the specified screening program over the default program (screening everyone at a specified age with medium sensitivity and specificity). Box: default program with 0.5 probability. High sensitivity/specificity = 80%, medium sensitivity/specificity = 60%, low sensitivity/specificity = 40%.

Figure 1 displays the likelihood that respondents would select a range of hypothetical screening programs that vary by risk factors, sensitivity, and specificity over a program in which eligibility is based on age with medium sensitivity and specificity. Overall, programs with the highest sensitivity were most likely to be preferred. For a given sensitivity and specificity, age and sex or genetics were always preferred over age alone and lifestyle risk scores. Within each value of sensitivity, respondents would accept a program with 20% lower specificity if the favored risk factors were used. For example, a program with a specificity of 60% using either age and sex or genetics was preferred over one with a specificity of 80% using either age alone or lifestyle risk scores.

**Figure 2:**
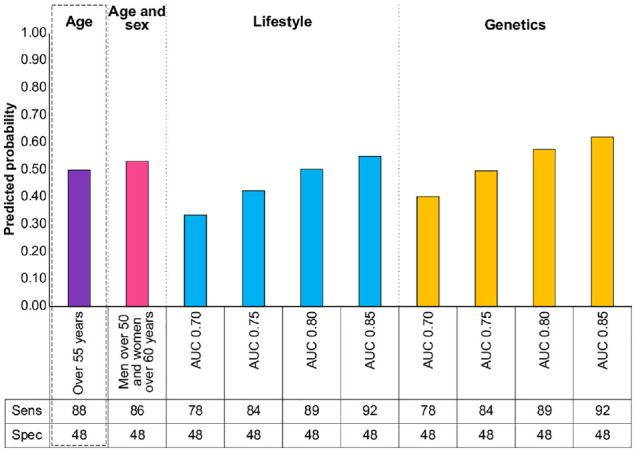
Predicted probabilities of choosing a specified screening program with fixed specificity compared with screening everyone at 55 y. Bars indicate the probability that respondents would choose the specified screening program over the default program (screening everyone at 55 y in a population aged 40 to 75 y, based on demographics and incidence of bowel and lung cancer in the United Kingdom). Box: default program with 0.5 probability. AUC: area under the receiver-operating characteristic curve.

Figure 2 shows the probability that respondents would opt for different risk-stratified screening programs in line with current risk prediction models. The age- and sex-based approach in this example is only slightly more acceptable than screening everyone from 55 y due to a similar sensitivity. A risk prediction model using genetics with an AUC ≥ 0.75 is needed for a program to be equally or more acceptable than screening everyone over of 55 y, while an AUC ≥ 0.80 is needed for a risk prediction model using lifestyle risk factors.

### Views on Attributes

Consistent with the regression analyses, the number of cancers detected and missed by a screening strategy (both based on underlying sensitivity) were perceived as the first or second most important attributes by 84.4% and 68.2% of 1,039 respondents, respectively (Supplementary Figure S2). Conversely, 71.2% respondents considered the number needing unnecessary follow-up (based on underlying specificity) as the least or second least important attribute when asked explicitly.

Analysis of the free-text comments showed that the respondents who found the DCE easy tended to prioritize certain attributes; those who found it difficult often attempted to weigh-up different attributes or the program as a whole (Supplementary Table S7). Respondents who took the latter approach described how it was not obvious which outcome(s) to prioritize and that “the benefits in some sections were often balanced by deficiencies in other areas” (57 y, female, mixed/multiple ethnicity, no cancer history).

Table 4 illustrates respondents’ views on individual attributes. Among those who prioritized specific attributes, most comments (*n* = 351/400, 87.8%) related to selecting the greatest number of cancers detected or the least number missed. These attributes were both synonymous with saving lives and more effective treatment to respondents: “that [reducing cancers missed] seemed like the best outcome of screening . . . make sure you catch as many people with cancer as early as possible” (36 y, male, White ethnicity, no cancer history). Some respondents used the number screened to calculate ratios or percentages of other attributes, although most appeared to consider the number screened itself important: “[I] based a vast majority on numbers invited, and some on the success” (30 y, female, White ethnicity, no cancer history) and “the more you screen the less you miss” (66 y, male, White ethnicity, no cancer history).

**Table 4:** Frequency and Illustrative Quotations of Respondents’ Views on the Attributes Included in the DCE^a,b^

Attribute	Number of Comments	Illustrative Quotations
Risk factors in model	18	•“. . . Although checking everyone would be ideal the current NHS cannot support it so trying to target factors that increase the likelihood of success seems a better method.” (23 y, female, White ethnicity, no cancer history, easy) •“When the factors differed i.e.: sex, genetic predisposition, lifestyle age etc., it made me think harder. Does someone who is genetically predisposed to cancer more ‘worthy’ of screening than say someone based on gender alone?” (48 y, female, Asian ethnicity, no cancer history, slightly difficult)
Number offered screening	120	•“The more people the better, whether detected or not, without it, you will never know.” (63 y, female, White ethnicity, history of cancer, very easy) •“Unnecessary screening can be harmful. . . . I tried to pick programs that minimized the number of people who had unnecessary follow-up, as well as minimizing the number who were offered screening at all.” (65 y, female, White ethnicity, history of cancer, slightly difficult)
Number of cancers detected or missed (lives saved)	435	•“For me, it was about the most lives saved—what detected the most cancers, and had the fewest undiagnosed cancers from those not tested.” (38 y, male, White ethnicity, no cancer history, slightly easy) •“Ultimately, I picked the ones which reduced the overall number of people with cancer being missed as a result. That seemed like the best outcome of screening is to make sure you catch as many people with cancer as early as possible.” (36 y, male, White ethnicity, no cancer history, easy) •“I mostly went on the number of cancers found but found it difficult to choose when the number of cancers missed was fairly high.” (55 y, female, White ethnicity, no cancer history, slightly difficult)
Number needing follow-up unnecessarily	54	•“. . . The more difficult part was the large difference in the number of people who might need a follow up which was unnecessary. It is harder to decide how many is too many in that scenario.” (60 y, female, White ethnicity, no cancer history, easy) •“Judged mainly on the number having a false-positive result and the number of cancers missed. False positives need to be evaluated and any screening needs to reduce this number as much as possible and all risks and benefits fully explained before informed consent can be obtained.” (62 y, female, White ethnicity, no cancer history, easy) •“. . . I also took into account the amount of unnecessary screenings that would be performed from a false positive, the NHS is under such financial, resource and staff shortage that any unnecessary treatment needs to be avoided.” (34 y, female, Asian ethnicity, no cancer history, slightly difficult)

a Thematic analysis of free-text comments given in response to the question, “Please tell us why you found choosing between the different programs easy or difficult.”

b Information presented in parentheses indicates demographics plus how easy or difficult individual respondents reported it was to select between programs in the discrete choice experiment.

When unnecessary follow-up was mentioned, this was mostly to describe why it had not been key in their decision making. Many respondents said they “would rather experience an unnecessary follow-up than potentially have a cancer diagnosis missed” (32 y, female, White ethnicity, no cancer history) because “medical appointments are not a massive burden when the end goal is preventing cancer” (24 y, male, White ethnicity, no cancer history). The resource requirement would therefore be acceptable: “I don’t want any unnecessary waste of money incurred by the health service but we have to save as many lives as we can” (53 y, female, White ethnicity, history of cancer).

Corresponding to the analyses above, risk factors were mentioned least frequently. When they were cited, respondents tended to believe that certain factors, particularly genetics, increased cancer risk, and so those affected should be screened. No one reported that a particular approach would be unacceptable.

## Discussion

It was clear that the sensitivity of the eligibility criteria, reflected by the proportion of cancers detected and not missed, was the respondents’ preferred characteristic, and the specificity of the criteria, reflected by the number of people offered screening or exposed to unnecessary follow-up, was much less important. The risk factors included were least important, but eligibility criteria based on age and sex or genetics were preferred over age alone or a lifestyle risk score, suggesting that risk stratification was acceptable. Although the strength of preferences sometimes varied according to certain characteristics, the order of importance of the attributes was consistent throughout. If and when risk stratification is introduced into screening programs, the increased benefit to those at higher risk is likely to appeal to public preferences more than the reduction in harms to those at lower risk. As such, there are likely to be more immediate benefits in terms of uptake from improving sensitivity, but helping the public to better understand the concept of specificity and its implications may support greater informed decision making.

A key consideration for national screening programs is capacity and resource limitations. Based on our data and the age distribution of the incidence of the 2 most common cancers to affect both men and women in the United Kingdom (bowel and lung cancer), to achieve a sufficient increase in sensitivity while screening the same proportion of the population, a risk prediction model would need to have very good discrimination (AUC ≥ 0.80 for phenotypic risk factors or ≥0.75 for genetic risk factors) to be more acceptable to the public than a fixed age for screening (in this case, 55 y). Several risk prediction models with comparable AUCs exist for bowel and lung cancers,^9,10^ but other cancers such as breast, kidney, and prostate tend to have AUCs < 0.75.^11–13^ Inclusion of biomarkers or previous screening results and larger genome-wide association studies are expected to result in meaningfully greater discrimination in the future. These factors are likely to be viewed as favorably by the public as genetics as they too would be based on nonsubjective, individual samples.^15^

The strength of preference that we observed for sensitivity for eligibility criteria is consistent with general public enthusiasm for cancer screening.^32^ The unimportance of unnecessary follow-up or screening harms is also consistent with willingness to accept overdetection^33^ and screening at low risk of cancer^34^ and acceptance of screening that unambiguously did not save lives but had the possibility of serious harms.^35^ These preferences were seen regardless of how people are selected for screening. Screening more people with the potential of identifying more cases of cancer was always preferred over attempts to reduce the number of people harmed by screening. This applies to current screening programs in which individuals are invited based on age, where the preference would be to offer screening at younger ages, as well as programs introducing risk stratification at the point of invitation. Additional benefits, particularly around informed decision making, could be gained by helping the public to better understand the value of specificity. This is particularly important for screening harms, which could be presented quantitatively, across the screening pathway and by population subgroup.^36^ It is also important to present harms and benefits using the comparable metrics.^37^ For example, we have shown that if specificity is presented only as additional, ultimately unnecessary, investigations that the public primarily consider to be a psychological harm, it is hard to trade off sensitivity if the public directly associate this with lives saved from cancer.

The relative unimportance of the risk factors used to determine eligibility and the perceived similarities between strategies were unexpected findings. Favoring genetics for determining risk has been observed elsewhere, where it was considered stable, objective, and free from the ethical considerations associated with lifestyle factors.^15,38^ Similarly, this may explain why using age and sex alone were more acceptable than adding lifestyle risk. These findings suggest the individual advantages and disadvantages of various risk factors are overshadowed by sensitivity when presented as a whole program, meaning that transparent and comprehensive communication with the public is important.^15^ This may have particular relevance for screening programs in which lifestyle factors are used for risk stratification, such as the lung cancer screening program, where eligibility is determined by smoking status.^39,40^

Our approach enabled us to present the sensitivity and specificity of the eligibility criteria and the corresponding modeled cancer outcomes to respondents in an accessible format while still quantifying tradeoffs between them in a way that is relevant to the research community. We presented participants with these outcomes in terms of anticipated number of people affected in a population, including an explanation of each attribute before the DCE, and used short descriptions within the questions themselves, which has been used effectively in similar studies.^41^ However, using outcomes calculated from sensitivity and specificity meant we needed to include 2 nonindependent attributes for each within the DCE. We covered all of the main harms of screening in the attributes based on specificity. However, the participants might not have appreciated the complete range of harms (physical effects, psychological effects, financial strain, and opportunity costs)^37^ because they were not explicit within the number of people invited to screening and needing follow-up unnecessarily. As for the attributes based on sensitivity, although it would have been interesting to know how the public considers a wider range of benefits and harms, it would not be feasible for respondents to learn about and weigh-up every aspect considered in policy decision making. This necessitated exclusion of attributes such as mortality and harms such as time and financial costs.

In our study, the qualitative element revealed some respondents’ misunderstanding, primarily equating sensitivity with mortality reduction directly. It is possible that including an attribute describing mortality reduction instead would have changed the outcomes. However, we do not think the conclusions would change significantly given the strength of respondents’ preferences for sensitivity and the public enthusiasm for cancer screening described above.^32–35^ The qualitative element additionally indicated that respondents’ underlying views identified in the DCE were consistent with their intuitive thoughts. We did not focus on eligibility for screening for a specified cancer type, meaning that we were able to identify universal principles although this necessitated providing generic information and may have had an impact on respondents’ views. In particular, unnecessary follow-up might have been more salient if a specific test was presented.

As in most online studies, the main limitations of our study relate to the sample. Use of an online recruitment platform enabled us to recruit a sample representative of the UK population with respect to age, sex, and ethnicity and large enough to explore differences between predefined subgroups. However, this sample may not be representative of the general population regarding other characteristics.^42^ Although we provided detailed written information, some respondents did not answer the example questions correctly, suggesting that some might have not understood all of the information.

## Conclusion

Sensitivity is the most important attribute of cancer screening eligibility criteria to the public. Moving from an age-based to a risk-stratified approach is therefore likely to be acceptable provided that sensitivity is high, even if specificity decreases somewhat. Communication to the public regarding new screening strategies that emphasize the expected improvements to the numbers of cancers detected or missed as a result will be most appealing but should still convey the importance of specificity to support informed decision making. While genetic risk predication models can afford to have a slightly lower discrimination than phenotypic models, sensitivity should be prioritized during the development of implementation strategies.

## Supplemental Material

sj-docx-1-mdm-10.1177_0272989X231155790 – Supplemental material for Public Preferences for Determining Eligibility for Screening in Risk-Stratified Cancer Screening Programs: A Discrete Choice Experiment Supplemental material, sj-docx-1-mdm-10.1177_0272989X231155790 for Public Preferences for Determining Eligibility for Screening in Risk-Stratified Cancer Screening Programs: A Discrete Choice Experiment by Rebecca A. Dennison, Lily C. Taylor, Stephen Morris, Rachel A. Boscott, Hannah Harrison, Sowmiya A. Moorthie, Sabrina H. Rossi, Grant D. Stewart and Juliet A. Usher-Smith in Medical Decision Making

sj-pdf-2-mdm-10.1177_0272989X231155790 – Supplemental material for Public Preferences for Determining Eligibility for Screening in Risk-Stratified Cancer Screening Programs: A Discrete Choice Experiment Supplemental material, sj-pdf-2-mdm-10.1177_0272989X231155790 for Public Preferences for Determining Eligibility for Screening in Risk-Stratified Cancer Screening Programs: A Discrete Choice Experiment by Rebecca A. Dennison, Lily C. Taylor, Stephen Morris, Rachel A. Boscott, Hannah Harrison, Sowmiya A. Moorthie, Sabrina H. Rossi, Grant D. Stewart and Juliet A. Usher-Smith in Medical Decision Making

## References

[1] Gilbert N . The pros and cons of screening. Nature. 2020;579:S2–4. 10.1038/d41586-020-00841-8 32214261

[2] Ponti A Anttila A Ronco G , et al. Cancer screening in the European Union. Report on the implementation of the council recommendation on cancer screening (second report, 2017). Available from: https://ec.europa.eu/health/system/files/2017-05/2017_cancerscreening_2ndreportimplementation_en_0.pdf. [Accessed 7 July, 2022].

[3] Brown KF Rumgay H Dunlop C , et al. The fraction of cancer attributable to modifiable risk factors in England, Wales, Scotland, Northern Ireland, and the United Kingdom in 2015. Br J Cancer. 2018;118:1130–41. 10.1038/s41416-018-0029-6 PMC5931106 29567982

[4] Anand P Kunnumakara AB Sundaram C , et al. Cancer is a preventable disease that requires major lifestyle changes. Pharm Res. 2008;25:2097–116. 10.1007/s11095-008-9661-9 PMC2515569 18626751

[5] Carr PR Weigl K Edelmann D , et al. Estimation of absolute risk of colorectal cancer based on healthy lifestyle, genetic risk, and colonoscopy status in a population-based study. Gastroenterology. 2020;159:129–38.e9. 10.1053/j.gastro.2020.03.016 PMC7387145 32179093

[6] Roos VH Mangas-Sanjuan C Rodriguez-Girondo M , et al. Effects of family history on relative and absolute risks for colorectal cancer: a systematic review and meta-analysis. Clin Gastroenterol Hepatol. 2019;7:2657–67.e9. 10.1016/j.cgh.2019.09.007 31525516

[7] Castle PE Katki HA . A risk-based framework to decide who benefits from screening. Nat Rev Clin Oncol. 2016;13:531–2. 10.1038/nrclinonc.2016.101 PMC5505517 27323876

[8] Shieh Y Eklund M Sawaya GF Black WC Kramer BS Esserman LJ . Population-based screening for cancer: hope and hype. Nat Rev Clin Oncol. 2016;13:550–65. 10.1038/nrclinonc.2016.50 PMC6585415 27071351

[9] Robbins HA Alcala K Swerdlow AJ , et al. Comparative performance of lung cancer risk models to define lung screening eligibility in the United Kingdom. Br J Cancer. 2021;124:2026–34. 10.1038/s41416-021-01278-0 PMC8184952 33846525

[10] McGeoch L Saunders CL Griffin SJ , et al. Risk prediction models for colorectal cancer incorporating common genetic variants: a systematic review. Cancer Epidemiol Biomarkers Prev. 2019;28:1580–93. 10.1158/1055-9965.EPI-19-0059 PMC7610631 31292139

[11] Aladwani M Lophatananon A Ollier W Muir K . Prediction models for prostate cancer to be used in the primary care setting: a systematic review. BMJ Open. 2020;10:e034661. 10.1136/bmjopen-2019-034661 PMC7371149 32690501

[12] Kim G Bahl M . Assessing risk of breast cancer: a review of risk prediction models. J Breast Imaging. 2021;3:144–55. 10.1093/jbi/wbab001 PMC7980704 33778488

[13] Harrison H Thompson RE Lin Z , et al. Risk prediction models for kidney cancer: a systematic review. Eur Urol Focus. 2021;7:1380–90. 10.1016/j.euf.2020.06.024 PMC8642244 32680829

[14] Dobrow MJ Hagens V Chafe R Sullivan T Rabeneck L . Consolidated principles for screening based on a systematic review and consensus process. Can Med Assoc J. 2018;190:E422–9. 10.1503/cmaj.171154 PMC5893317 29632037

[15] Dennison RA Boscott RA Thomas R , et al. A community jury study exploring the public acceptability of using risk stratification to determine eligibility for cancer screening. Health Expect. 2022;25:1789–806. 10.1111/hex.13522 PMC9327868 35526275

[16] Meisel SF Rahman B Side L , et al. Genetic testing and personalized ovarian cancer screening: a survey of public attitudes. BMC Womens Health. 2016;16:46. 27460568 10.1186/s12905-016-0325-3 PMC4962369

[17] Fisher BA Wilkinson L Valencia A . Women’s interest in a personal breast cancer risk assessment and lifestyle advice at NHS mammography screening. J Public Health. 2016;39:113–21. 10.1093/pubmed/fdv211 PMC5356472 26834190

[18] Wheeler JCW Keogh L Sierra MA , et al. Heterogeneity in how women value risk-stratified breast screening. Genet Med. 2022;24:146–56. 10.1016/j.gim.2021.09.002 34906505

[19] Hall R Medina-Lara A Hamilton W Spencer AE . Attributes used for cancer screening discrete choice experiments: a systematic review. Patient. 2022;15:269–85. 10.1007/s40271-021-00559-3 34671946

[20] Bridges JFP Hauber AB Marshall D , et al. Conjoint analysis applications in health—a checklist: a report of the ISPOR good research practices for conjoint analysis task force. Value Health. 2011;14:403–13. 10.1016/j.jval.2010.11.013 21669364

[21] Reed Johnson F Lancsar E Marshall D , et al. Constructing experimental designs for discrete-choice experiments: report of the ispor conjoint analysis experimental design good research practices task force. Value Health. 2013;6:3–13. 10.1016/j.jval.2012.08.2223 23337210

[22] Publishers Audience Measurement Company Ltd (PAMCo). Interview and questionnaire. Available from: https://pamco.co.uk/how-it-all-works/interview-and-questionnaire. [Accessed 7 July, 2022].

[23] Smits SE McCutchan GM Hanson JA Brain KE . Attitudes towards lung cancer screening in a population sample. Health Expect. 2018;21:1150–8. 10.1111/hex.12819 PMC6250881 30085384

[24] Schwartz LM Woloshin S Black WC Welch HG . The role of numeracy in understanding the benefit of screening mammography. Ann Intern Med. 1997;127:966–72. 10.7326/0003-4819-127-11-199712010-00003 9412301

[25] Simon AE Forbes LJL Boniface D , et al.; ICBP Module 2 Working Group, ICBP Programme Board and Academic Reference Group. An international measure of awareness and beliefs about cancer: development and testing of the ABC. BMJ Open. 2012;2:e001758. 10.1136/bmjopen-2012-001758 PMC3547316 23253874

[26] Lerman C Trock B Rimer BK Boyce A Jepson C Engstrom PF . Psychological and behavioral implications of abnormal mammograms. Ann Intern Med. 1991;114:657–61. 10.7326/0003-4819-114-8-657 2003712

[27] Anwyl-Irvine AL Massonnié J Flitton A Kirkham N Evershed JK . Gorilla in our midst: an online behavioral experiment builder. Behav Res Methods. 2020;52:388–407. 31016684 10.3758/s13428-019-01237-x PMC7005094

[28] Lancsar E Louviere J . Conducting discrete choice experiments to inform healthcare decision making. Pharmacoeconomics. 2008;26:661–77. 10.2165/00019053-200826080-00004 18620460

[29] Hauber AB González JM Groothuis-Oudshoorn CGM , et al. Statistical methods for the analysis of discrete choice experiments: a report of the ISPOR Conjoint analysis good research practices task force. Value Health. 2016;19:300–15. 10.1016/j.jval.2016.04.004 27325321

[30] Cancer Research UK. Statistics by cancer type. Available from: www.cancerresearchuk.org/health-professional/cancer-statistics/statistics-by-cancer-type. [Accessed 7 July, 2022].

[31] Office of National Statistics. Estimates of the population for the UK, England and Wales, Scotland and Northern Ireland dataset. Available from: www.ons.gov.uk/peoplepopulationandcommunity/populationandmigration/populationestimates/datasets/populationestimatesforukenglandandwalesscotlandandnorthernireland. [Accessed 8 February, 2022].

[32] Waller J Osborne K Wardle J . Enthusiasm for cancer screening in Great Britain: a general population survey. Br J Cancer. 2015;112:562–6. 10.1038/bjc.2014.643 PMC4453657 25535731

[33] van den Bruel A Jones C Yang Y Oke J Hewitson P . People’s willingness to accept overdetection in cancer screening: population survey. BMJ. 2015;350:h980. 10.1136/bmj.h980 PMC4356995 25736617

[34] Banks J Hollinghurst S Bigwood L Peters TJ Walter FM Hamilton W . Preferences for cancer investigation: a vignette-based study of primary-care attendees. Lancet Oncol. 2014;15:232–40. 10.1016/S1470-2045(13)70588-6 24433682

[35] Scherer LD Valentine KD Patel N Baker SG Fagerlin A . A bias for action in cancer screening? J Exp Psychol Appl. 2019;25:149–61. 10.1037/xap0000177 30024212

[36] Kamineni A Doria-Rose VP Chubak J , et al. Evaluation of harms reporting in US cancer screening guidelines. Ann Intern Med. 2022;175:1582–90. 10.7326/M22-1139 PMC9903969 36162112

[37] Harris RP Sheridan SL Lewis CL , et al. The harms of screening: a proposed taxonomy and application to lung cancer screening. JAMA Intern Med. 2014;174:281–5. 10.1001/jamainternmed.2013.12745 24322781

[38] Henneman L Timmermans DR Bouwman CM Cornel MC Meijers-Heijboer H . ‘A low risk is still a risk’: exploring women’s attitudes towards genetic testing for breast cancer susceptibility in order to target disease prevention. Public Health Genomics. 2011;14:238–47. 10.1159/000276543 20090298

[39] Krist AH Davidson KW Mangione CM , et al. Screening for lung cancer: US preventive services task force recommendation statement. JAMA. 2021;325:962–70. 10.1001/jama.2021.1117 33687470

[40] Cancer Research UK. Lung health checks. Available from: https://www.cancerresearchuk.org/about-cancer/lung-cancer/getting-diagnosed/lung-health-checks. [Accesssed 10 January, 2023].

[41] Ghanouni A Halligan S Taylor SA , et al. Evaluating patients’ preferences for type of bowel preparation prior to screening CT colonography: convenience and comfort versus sensitivity and specificity. Clin Radiol. 2013;68:1140–5. 10.1016/j.crad.2013.06.018 23948662

[42] Palan S Schitter C . Prolific.ac—a subject pool for online experiments. J Behav Exp Finance. 2018;17:22–7.

