# Supplementary material for: Public Preferences for Determining Eligibility for Screening in Risk-Stratified Cancer Screening Programs: A Discrete Choice Experiment
Source: Med Decis Making. 2023 Feb 14;43(3):374–86. doi: 10.1177/0272989X231155790 (PMC10021112; doi:10.1177/0272989X231155790)
Supplement: sj-pdf-2-mdm-10.1177_0272989X231155790 – Supplemental material for Public Preferences for Determining Eligibility for Screening in Risk-Stratified Cancer Screening Programs: A Discrete Choice Experiment [file sj-pdf-2-mdm-10.1177_0272989X231155790.pdf]

Public preferences for determining eligibility for screening in risk-stratified cancer screening programmes: A discrete choice experiment

Supplementary material

Supplementary File 1: EXPRESS-DCE questionnaire. 2

Supplementary Table 1: Conversion of sensitivity and specificity of the eligibility criteria (underlying attributes) into the attributes displayed to participants in the DCE 10

Supplementary Table 2: Participants' views on cancer and screening. 11

Supplementary Table 3: Numeracy, DCE checks and evaluation. 13

Supplementary Table 4: Conditional logit regression using displayed attributes plus relative importance of attributes. 14

Supplementary Table 5: Subgroup analyses of conditional logit regression using underlying attributes (according to binary groups by demographics, numeracy, and views on cancer and screening). 15

Supplementary Table 6: Sensitivity analysis – Conditional logit regression using underlying attributes plus relative importance of attributes comparing participants who passed and failed the rationality check. 18

Supplementary Table 7: Frequency and illustrative quotations of respondents' approaches to selecting between programmes in the DCE. 19

Supplementary Figure 1: Ease of completing the DCE by study arm. 20

Supplementary Figure 2: Participants' ranking of attributes included in the DCE. 21

Supplementary File 1: EXPRESS-DCE questionnaire.

Note: This content was delivered online. Participants did not see the headings in shaded boxes or the references.

**Part A. Introduction and consent**	
**Part B. Demographics and views towards cancer**	
**1. Demographics**	
Demographic information *In this first section we would like to ask you a few questions about yourself. These questions allow us to make sure we are including people from a range of different backgrounds and see if different groups of people have different views. We will not be able to identify you from your answers.*	
How old are you?	[free text] years
What is your sex? (A question about gender identity will follow)	• Female<br>• Male
Is the gender you identify with the same as your sex registered at birth?	• Yes<br>• No (Enter gender identity)
What is your ethnic group?<br>Choose one option that best describes your ethnic group or background.	• Asian/Asian British<br>• Black/African/Caribbean/Black British<br>• Mixed/Multiple ethnic group<br>• White<br>• Other, please describe______
What is your highest education level?	• Finished school at or before the age of fifteen<br>• Completed GCSEs, O Levels or equivalent<br>• Completed A Levels or equivalent<br>• Completed further education but not a degree<br>• Completed a Bachelor's degree / Master's degree / PhD<br>• Other (please specify) ______
Which of the following best describes the main income earner's main/most recent job?<br>This could be you: the main income earner is the person in your household with the largest income.<br>If the main income earner is retired and has a pension, please answer for their most recent occupation. If the main income earner is not in paid employment but has been out of work for less than 6 months, please answer for their most recent occupation.[1,2]	• Business owner – responsible for 1-4 employees<br>• Business owner – responsible for 5-24 employees<br>• Business owner – responsible for 25+ employees<br>• Top/senior manager in large organisation; qualified senior professional e.g. main board director, senior civil servant, headteacher, partner in professional practice, surgeon<br>• Middle manager or executive in large organisation; senior manager of small organisation; qualified professional (no senior management responsibility) e.g. department manager, teacher, engineer, accountant, doctor, manager of small building firm<br>• Non-management office role; middle manager in small organisation; qualified nurse; performing artist e.g. secretary, personal assistant, clerical worker, office worker, call centre agent, salesperson, nurse or nursery nurse, police constable/sergeant, actor, musician, sportsperson<br>• Manual worker - manager – responsible for 25+ employees

	• Manual worker - manager – responsible for 1-24 employees<br>• Skilled manual worker (no responsibility for other employees) e.g. HGV driver, rain/bus/ambulance driver, chef, hairdresser, mechanic, plumber, bricklayer, carpenter, painter, electrician, caterer, specialised machinery operator, fire-fighter, pub/bar worker<br>• Unskilled or semi-skilled manual worker (no responsibility for other employees) e.g. farm worker, cleaner, postal worker, van driver, care worker, waiter, taxi driver, shop assistant, apprentice/trainee in skilled trade<br>• Casual worker or no regular income<br>• Full time student
What is your tobacco smoking status?	• Never smoked cigarettes or cigars<br>• Used to smoke cigarettes or cigars<br>• Smoke up to 20 cigarettes or cigars per day<br>• Smoke 20 or more cigarettes or cigars per day
How would you describe your weight?	• Underweight<br>• About the right weight<br>• Slightly overweight<br>• Very overweight
Have you ever had cancer?	• Yes<br>• No
Have your parents or any brothers or sisters ever had cancer?	• Yes<br>• No<br>• Don’t know
Has anyone close to you (e.g. a partner or close friend) ever had cancer?	• Yes<br>• No<br>• Don’t know
**2. Numeracy**	
Numeracy<br>*As the information we will be giving you about cancer screening includes numbers, we would like to know how you answer the following questions.*	
Imagine we flip a fair coin 1,000 times.<br>• What is your best guess at how many times the coin would come up heads in 1,000 flips?	[free text] times out of 1,000
In the UK National Lottery®, the chance of winning a £10 prize is 1%.<br>• What is your best guess at how many people would win a £10 prize if 1,000 people each bought a single ticket to UK National Lottery®?	[free text] person(s) out of 1,000.

In the EuroMillions® Lottery, the chance of winning a car is 1 in 1,000.<br>• What percent of the EuroMillions® tickets win a car?[3]	[free text] %.
**3. Thoughts and beliefs about cancer**	
Thoughts and beliefs about cancer<br>*The next section asks about your thoughts and beliefs about cancer. Please answer as honestly as you can as this will help us with our analysis. The questions are about YOUR opinion and so there is not a correct answer.*	
Below are some statements that are sometimes made about cancer. For each of these statements how much do you agree or disagree with them?[2,4]<br>• These days, many people with cancer can expect to continue with normal activities and responsibilities.<br>• Most cancer treatment is worse than the cancer itself.<br>• I would NOT want to know if I have cancer.<br>• Cancer can often be cured.<br>• Going to the doctor as quickly as possible after noticing a symptom of cancer could increase the chances of surviving.<br>• Some people think a diagnosis of cancer is a death sentence.	• Strongly agree<br>• Agree<br>• Neither disagree nor agree<br>• Disagree<br>• Strongly disagree
How likely do you think is it that you will get cancer at some point in the next 10 years?	• Extremely likely<br>• Moderately likely<br>• Slightly likely<br>• Neither likely nor unlikely<br>• Slightly unlikely<br>• Moderately unlikely<br>• Extremely unlikely
For each of the following statements, select the option that best applies to you:[5]<br>• During the past month, how often have you thought about your own chances of getting cancer?<br>• During the past month, how often have thoughts about your chances of getting cancer affected your mood?<br>• During the past month, how often have thoughts about your chances of getting cancer affected your ability to perform your daily activities?	• Not at all<br>• Rarely<br>• Sometimes<br>• Often<br>• A lot

Do you think that benefits of cancer screening outweigh the possible side effects, potential harms and inconvenience?
Select all that apply.

- Yes, for everyone
- No, for everyone
- It depends on your age and sex
- It depends on the type of cancer
- It depends on how you feel about cancer
- It depends on how you feel about screening tests

Part C. Conjoint-analysis tasks

4. Explanation of each of the attributes and levels, and example

Instructions
We will now present you with a series of nine questions with two different strategies for cancer screening programmes in each question. For each question, you will be asked to select which programme you think is best. Again, the questions are about YOUR opinion and so there is not a correct answer. You might find some questions to be more straightforward than others. They will help us to understand which outcomes of the programme people think are most important.

We will provide the following information about each programme:

<u>The population</u>

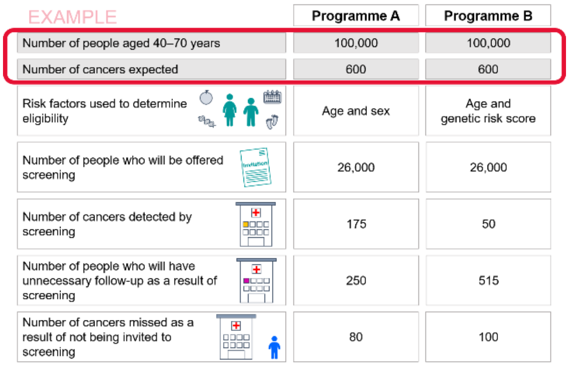

EXAMPLE	Programme A	Programme B
Number of people aged 40–70 years	100,000	100,000
Number of cancers expected	600	600
Risk factors used to determine eligibility	Age and sex	Age and genetic risk score
Number of people who will be offered screening	26,000	26,000
Number of cancers detected by screening	175	50
Number of people who will have unnecessary follow-up as a result of screening	250	515
Number of cancers missed as a result of not being invited to screening	80	100

The figure shows two possible cancer screening programmes and the outcomes for a population of 100,000 people aged 40 to 70 years.

For the purpose of this study, the number of people with cancer in the population of 100,000 people will be the same in each scenario (600 people with cancer).

Similarly, the same screening test will be used so the burden on each person tested, the cost per test and the accuracy of the test is the same. The impact of detecting the cancer through screening on survival will also be assumed to be constant.

<u>Risk factors used to determine eligibility for screening</u>

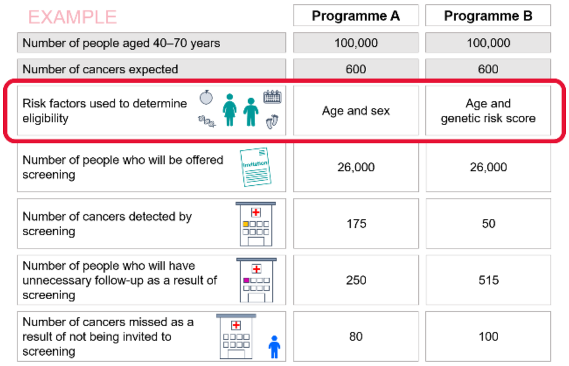

EXAMPLE	Programme A	Programme B
Number of people aged 40–70 years	100,000	100,000
Number of cancers expected	600	600
Risk factors used to determine eligibility	Age and sex	Age and genetic risk score
Number of people who will be offered screening	26,000	26,000
Number of cancers detected by screening	175	50
Number of people who will have unnecessary follow-up as a result of screening	250	515
Number of cancers missed as a result of not being invited to screening	80	100

Only people with certain characteristics will be sent an invitation for screening. This is because not everyone has the same risk of developing cancer. The approaches for determining eligibility for screening using different risk factors include:

1. Age – *Cancer is more common in older people, although being younger also does not mean someone is completely without risk of getting cancer. Everyone over a certain age could be invited for screening.*

2. Age and sex – *Some cancers are more common in males than females, or vice versa. All males over a certain age could be invited for screening, or males over a certain age and females over a different age.*

3. Age, sex and other lifestyle risk factors such as weight, smoking and ethnicity – *Just as cancer is more common in older people, other characteristics mean that some people are more likely to develop certain cancers than other people. For example, people who are overweight and those who smoke often have a higher risk. This does not mean that all people who are overweight or smoke develop cancer or that people who are not overweight and do not smoke do not develop cancer. It just means that on average people who are overweight or smoke are more likely to develop some types of cancer than people who are not overweight and do not smoke.*

4. Age and genetic risk – *Lastly, some people have certain genes that put them at higher risk of cancer, particularly as they get older.*

Information about the risk factors would need to be collected for the risk assessment. This could be done through the GP records or questionnaires. A cheek swab or finger prick blood test could be used to assess genetics.

Experts have developed calculators to estimate how likely an individual is to develop certain cancers. The calculators are based on scientific research studies and use information about these risk factors to identify when someone should first have cancer screening. As with all of these approaches, they are not 100% reliable. Someone who is estimated to be at higher risk based on the calculator is not destined to get cancer. A low risk estimate also does not mean someone is completely without risk of getting cancer.

In the questions that follow, we won't explain exactly how the risk factors have been used to decide when to invite someone to screening. For example, using the risk factors 'age and sex' could mean screening:

- *women over 50 years old and men over 60 years old, or*
- *women over 45 years old and men over 40 years old, or*
- *something else.*

<u>Number of people who will be offered screening</u>

EXAMPLE	Programme A	Programme B
Number of people aged 40–70 years	100,000	100,000
Number of cancers expected	600	600
Risk factors used to determine eligibility	Age and sex	Age and genetic risk score
Number of people who will be offered screening	26,000	26,000
Number of cancers detected by screening	175	50
Number of people who will have unnecessary follow-up as a result of screening	250	515
Number of cancers missed as a result of not being invited to screening	80	100

Depending on how these risk factors are used, different numbers of people will be invited to cancer screening. For example, more people will be offered screening if people over 50 years old are invited than if people over 60 years old are invited because there are more people alive over 50 than over 60.

<u>Number of cancers detected by screening</u>

EXAMPLE	Programme A	Programme B
Number of people aged 40–70 years	100,000	100,000
Number of cancers expected	600	600
Risk factors used to determine eligibility	Age and sex	Age and genetic risk score
Number of people who will be offered screening	26,000	26,000
Number of cancers detected by screening	175	50
Number of people who will have unnecessary follow-up as a result of screening	250	515
Number of cancers missed as a result of not being invited to screening	80	100

People with a positive screening test will be offered further diagnostic tests for cancer such as a biopsy or scan. Cancer will be detected in some of these people. Many of those will then be offered treatment and their lives could be saved.

<u>Number of people who will have unnecessary follow-up as a result of screening</u>

EXAMPLE	Programme A	Programme B
Number of people aged 40–70 years	100,000	100,000
Number of cancers expected	600	600
Risk factors used to determine eligibility	Age and sex	Age and genetic risk score
Number of people who will be offered screening	26,000	26,000
Number of cancers detected by screening	175	50
Number of people who will have unnecessary follow-up as a result of screening	250	515
Number of cancers missed as a result of not being invited to screening	80	100

No screening test is perfect, and some people who have a positive screening test don't actually have cancer. They too will be offered further diagnostic tests for cancer such as a biopsy or scan. This can cause anxiety and pain or discomfort.

<u>Number of cancers missed as a result of not being invited to screening</u>

EXAMPLE	Programme A	Programme B
Number of people aged 40–70 years	100,000	100,000
Number of cancers expected	600	600
Risk factors used to determine eligibility	Age and sex	Age and genetic risk score
Number of people who will be offered screening	26,000	26,000
Number of cancers detected by screening	175	50
Number of people who will have unnecessary follow-up as a result of screening	250	515
Number of cancers missed as a result of not being invited to screening	80	100

Just as most people with risk factors will not actually develop cancer, some people without risk factors will develop cancer. They won't have been offered screening because they weren't considered to be at high risk. They may not be diagnosed until they develop symptoms and treatment may be less successful if the cancer has been picked up later.

For the purpose of this study, the number of people with cancer in the population is the same in each scenario (600 people with cancer). Similarly, the same screening test is used so the burden on each person tested, the cost per test and the accuracy of the test is the same. The impact of detecting the cancer through screening on survival is also assumed to be constant.

On the next page, we will ask you three questions about this example so that we can check that we've explained these ideas clearly.

Once you click 'Next', you won't be able to come back to these instructions.

Example

Imagine that there were 100,000 people aged 40–70 years in a population. 600 people were expected to have a particular type of cancer.

The figure shows two possible screening programmes for this cancer and the outcomes for the population.

EXAMPLE	Programme A	Programme B
Number of people aged 40–70 years	100,000	100,000
Number of cancers expected	600	600
Risk factors used to determine eligibility	Age and sex	Age and genetic risk score
Number of people who will be offered screening	26,000	26,000
Number of cancers detected by screening	175	50
Number of people who will have unnecessary follow-up as a result of screening	250	515
Number of cancers missed as a result of not being invited to screening	80	100

Looking at the example, please respond true or false to the following statements:<br>• More people would be invited to screening in Programme A<br>• More people in Programme B would have unnecessary follow-up as a result of screening<br>• The number of cancers detected is the same in both programmes	• True<br>• False

5. Conjoint-analysis task/DCE

The figure shows two possible cancer screening programmes and the outcomes for a population of 100,000 people aged 40–70 years.

EXAMPLE	Programme A	Programme B
Number of people aged 40–70 years	100,000	100,000
Number of cancers expected	600	600
Risk factors used to determine eligibility	Age	Age and lifestyle risk score
Number of people who will be offered screening	45,800	26,000
Number of cancers detected by screening	175	52
Number of people who will have unnecessary follow-up as a result of screening	251	515
Number of cancers missed as a result of not being invited to screening	77	101

Which programme do you think is best?	• Programme A<br>• Programme B

[This question was repeated nine times with different cancer screening programmes, with participants randomised 1:1:1:1 to:

- *Question block 1, order 1*
- *Question block 1, order 2*
- *Question block 2, order 1*
- *Question block 2, order 2]*

6. Evaluation

Please tell us how easy or difficult you found choosing between the different programmes.	• Very easy<br>• Easy<br>• Difficult<br>• Very difficult
Please tell us why you found choosing between the different programmes easy or difficult.	[Free text]
Please rank the details about the programmes in order of their importance to you with 1 being the aspect you consider *most important* and 5 being the aspect you consider *least important*.	*Rank:*<br>• Risk factors used to determine eligibility<br>• Number of people who will be offered screening<br>• Number of cancers detected by screening<br>• Number of people who will have unnecessary follow-up as a result of screening<br>• Number of cancers missed as a result of not being invited to screening

References

1. Publishers Audience Measurement Company Ltd (PAMCo) Interview and questionnaire. Available from: https://pamco.co.uk/how-it-all-works/interview-and-questionnaire. Last accessed 6 July 2022.
2. Smits SE, McCutchan GM, Hanson JA, Brain KE. Attitudes towards lung cancer screening in a population sample. Health Expectations 2018; 21: 1150–8.
3. Schwartz LM, Woloshin S, Black WC, Welch HG. The role of numeracy in understanding the benefit of screening mammography. Annals of Internal Medicine 1997; 127: 966–72.
4. Simon AE, Forbes LJL, Boniface D, et al. An international measure of awareness and beliefs about cancer: Development and testing of the ABC. BMJ Open 2012; 2: e001758.
5. Lerman C, Trock B, Rimer BK, Boyce A, Jepson C, Engstrom PF. Psychological and behavioral implications of abnormal mammograms. Annals of internal medicine 1991; 114: 657–61.

Supplementary Table 1: Conversion of sensitivity and specificity of the eligibility criteria (underlying attributes) into the attributes displayed to participants in the DCE.

The population (100,000 people aged 40-70 years), expected number of cases of cancer in the population (600 cases), and sensitivity and specificity of the screening test (53% and 99%, respectively) were held constant. These values were estimated from estimates of incidence of common cancers in the UK.

	Expected	Eligibility for screening							Performance of screening test in those screened					
Population	cancer cases	Sens of eligibility	Spec of eligibility	True positives	**False negatives[1]**	False positives	True negatives	**Total screened[2*]**	Sens of test	Spec of test	**True positives[3]**	False negatives	**False positives[4]**	True negatives
100,000	600	40	25	240	**360**	74,550	24,850	**74,950**	53	99	**127**	113	**746**	73,805
100,000	600	40	55	240	**360**	44,730	54,670	**45,120**	53	99	**127**	113	**447**	44,283
100,000	600	40	70	240	**360**	29,820	69,580	**30,210**	53	99	**127**	113	**298**	29,522
100,000	600	40	80	240	**360**	19,880	79,520	**20,270**	53	99	**127**	113	**199**	19,681
100,000	600	55	25	330	**270**	74,550	24,850	**45,120**	53	99	**175**	155	**746**	73,805
100,000	600	55	55	330	**270**	44,730	54,670	**45,120**	53	99	**175**	155	**447**	44,283
100,000	600	55	70	330	**270**	29,820	69,580	**30,210**	53	99	**175**	155	**298**	29,522
100,000	600	55	80	330	**270**	19,880	79,520	**20,270**	53	99	**175**	155	**199**	19,681
100,000	600	75	25	450	**150**	74,550	24,850	**74,950**	53	99	**239**	212	**746**	73,805
100,000	600	75	55	450	**150**	44,730	54,670	**45,120**	53	99	**239**	212	**447**	44,283
100,000	600	75	70	450	**150**	29,820	69,580	**30,210**	53	99	**239**	212	**298**	29,522
100,000	600	75	80	450	**150**	19,880	79,520	**20,270**	53	99	**239**	212	**199**	19,681
100,000	600	90	25	540	**60**	74,550	24,850	**74,950**	53	99	**286**	254	**746**	73,805
100,000	600	90	55	540	**60**	44,730	54,670	**45,120**	53	99	**286**	254	**447**	44,283
100,000	600	90	70	540	**60**	29,820	69,580	**30,210**	53	99	**286**	254	**298**	29,522
100,000	600	90	80	540	**60**	19,880	79,520	**20,270**	53	99	**286**	254	**199**	19,681

[1]Indicates the displayed attribute *number of cancers missed as a result of not being invited to screening*
[2]Indicates the displayed attribute *number of people who will be offered screening*
[3]Indicates the displayed attribute *number of cancers detected by screening*
[4]Indicates the displayed attribute *number of people who will have unnecessary follow-up as a result of screening*

*These calculations resulted in levels across a small range so were replaced with one average.

Sens: sensitivity; spec: specificity

Cancer Research UK. www.cancerresearchuk.org/health-professional/cancer-statistics/statistics-by-cancer-type. Date accessed: 07 July 2022.

Supplementary Table 2: Participants' views on cancer and screening.

	Whole population (n=1,172)	Passed rationality check (n=1,084)
**How likely do you think is it that you will get cancer at some point in the next 10 years?**		
Extremely unlikely	45 (3.8)	43 (4.0)
Moderately unlikely	108 (9.2)	102 (9.4)
Slightly unlikely	95 (8.1)	82 (7.6)
Neither likely nor unlikely	278 (23.7)	255 (23.5)
Slightly likely	351 (30.0)	327 (30.2)
Moderately likely	249 (21.3)	232 (21.4)
Extremely likely	46 (3.9)	43 (4.0)
**Beliefs about cancer**		
These days, many people with cancer can expect to continue with normal activities and responsibilities		
Strongly agree	73 (6.2)	68 (6.3)
Agree	690 (58.9)	644 (59.4)
Neither disagree nor agree	216 (18.4)	196 (18.1)
Disagree	180 (15.4)	163 (15.0)
Strongly disagree	13 (1.1)	13 (1.2)
Most cancer treatment is worse than the cancer itself		
Strongly agree	35 (3.0)	33 (3.0)
Agree	321 (27.4)	290 (26.8)
Neither disagree nor agree	409 (34.9)	378 (34.9)
Disagree	350 (29.9)	328 (30.3)
Strongly disagree	57 (4.9)	55 (5.1)
I would not want to know if I have cancer		
Strongly agree	8 (0.7)	8 (0.7)
Agree	52 (4.4)	49 (4.5)
Neither disagree nor agree	102 (8.7)	89 (8.2)
Disagree	407 (34.7)	376 (34.7)
Strongly disagree	603 (51.5)	562 (51.8)
Cancer can often be cured		
Strongly agree	152 (13.0)	140 (12.9)
Agree	704 (60.1)	654 (60.3)
Neither disagree nor agree	204 (17.4)	187 (17.3)
Disagree	99 (8.5)	91 (8.4)
Strongly disagree	13 (1.1)	12 (1.1)
Going to the doctor as quickly as possible after noticing a symptom of cancer could increase the chances of surviving		
Strongly agree	917 (78.2)	860 (79.3)
Agree	232 (19.8)	204 (18.8)
Neither disagree nor agree	15 (1.3)	13 (1.2)
Disagree	3 (0.3)	3 (0.3)
Strongly disagree	5 (0.4)	4 (0.4)
Some people think a diagnosis of cancer is a death sentence		
Strongly agree	257 (21.9)	242 (22.3)
Agree	774 (66.0)	714 (65.9)
Neither disagree nor agree	79 (6.7)	70 (6.5)
Disagree	44 (3.8)	42 (3.9)
Strongly disagree	18 (1.5)	16 (1.5)
**Cancer worry**		
During the past month, how often…		
…have you thought about your own chances of getting cancer?		
Not at all	299 (25.5)	277 (25.6)
Rarely	363 (31.0)	341 (31.5)
Sometimes	347 (29.6)	321 (29.6)
Often	133 (11.4)	117 (10.8)
A lot	30 (2.6)	28 (2.6)
…have thoughts about your chances of getting cancer affected your mood?		
Not at all	556 (47.4)	519 (47.9)
Rarely	342 (29.2)	317 (29.2)
Sometimes	206 (17.6)	186 (17.2)
Often	54 (4.6)	49 (4.5)
A lot	14 (1.2)	13 (1.2)
…have thoughts about your chances of getting cancer affected your ability to perform your daily activities?		
Not at all	851 (72.6)	795 (73.3)
Rarely	219 (18.7)	200 (18.5)
Sometimes	84 (7.2)	74 (6.8)
Often	10 (0.9)	7 (0.6)
A lot	8 (0.7)	8 (0.7)

Do you think that benefits of cancer screening outweigh the possible side effects, potential harms and inconvenience?		
Yes, for everyone	680 (58.0)	632 (58.3)
No, for everyone	24 (2.1)	24 (2.2)
It depends on:	466 (39.8)	426 (39.3)
your age and sex	270 (23.0)	247 (22.8)
the type of cancer	317 (27.1)	295 (27.2)
how you feel about cancer	158 (13.5)	148 (13.7)
how you feel about screening tests	179 (15.3)	170 (15.7)

N (%) reported.

Supplementary Table 3: Numeracy, DCE checks and evaluation.

	Whole population (n=1,172)	Passed rationality check (n=1,084)
**Number of correct numeracy questions (out of 3)**		
0	37 (3.2)	32 (3.0)
1	187 (16.0)	154 (14.2)
2	408 (34.8)	379 (35.0)
3	540 (46.1)	519 (47.9)
**Number of correct DCE understanding questions (out of 3)**		
0	16 (1.4)	13 (1.2)
1	181 (15.4)	157 (14.5)
2	564 (48.1)	522 (48.2)
3	411 (35.1)	392 (36.2)
**DCE evaluation – ease of selection**		
Very easy	51 (4.4)	50 (4.6)
Easy	236 (20.1)	224 (20.7)
Slightly easy	305 (26.0)	290 (26.8)
Slightly difficult	414 (35.3)	378 (34.9)
Difficult	140 (12.0)	121 (11.2)
Very difficult	24 (2.1)	19 (1.8)
Missing	2 (0.2)	2 (0.2)

N (%) reported.

Supplementary Table 4: Conditional logit regression using displayed attributes plus relative importance of attributes.

As the displayed attributes were not independent, this analysis was separated into two so that only one measure of sensitivity and specificity, as indicated, was included in each model.

Attribute	Coefficient (95% CI)	P value for coefficient	Relative importance (%)
**Model A**			
Risk factors in model			5.9
Age	Reference		
Age and sex	0.239 (0.136–0.342)	<0.001	
Age, sex and lifestyle risk score	-0.051 (-0.168–0.066)	0.394	
Age and genetic risk score	0.242 (0.152–0.332)	<0.001	
Number of cancers detected*	0.020 (0.019–0.021)	<0.001	82.0
Number needing follow-up unnecessarily**	-0.001 (-0.001–-0.001)	<0.001	12.1
**Model B**			
Risk factors in model			7.4
Age	Reference		
Age and sex	0.255 (0.149–0.360)	<0.001	
Age, sex and lifestyle risk score	0.026 (-0.091–0.144)	0.659	
Age and genetic risk score	0.253 (0.162–0.343)	<0.001	
Number offered screening**	0.000 (0.000–0.000)***	<0.001	11.3
Number of cancers missed*	-0.011 (-0.011–-0.010)	<0.001	81.3

N=1,084; Number of observations=17,344; pseudo R^2= 0.3953and 0.3931. CI: confidence interval.

*Calculated from the sensitivity of the eligibility criteria.
**Calculated from the specificity of the eligibility criteria.

***-8.01x10^{-6} (-9.80x10^{-6}–-6.23x10^{-6}).

Supplementary Table 5: Subgroup analyses of conditional logit regression using underlying attributes (according to binary groups by demographics, numeracy, and views on cancer and screening).

A. Age

	≤45 years (n=538)		>45 years (n=546)		
Attribute	Coefficient (95% CI)	P value for coefficient	Coefficient (95% CI)	P value for coefficient	P value for difference
**Risk factors in model**					
Age	Ref		Ref		
Age and sex	0.268 (0.125–0.411)	<0.001	0.207 (0.058–0.356)	0.007	0.556
Age, sex and lifestyle risk score	-0.024 (-0.187–0.139)	0.773	-0.079 (-0.248–0.090)	0.362	0.655
Age and genetic risk score	0.277 (0.153–0.402)	<0.001	0.201 (0.071–0.332)	0.003	0.417
**Sensitivity**	0.061 (0.058–0.064)	<0.001	0.066 (0.062–0.070)	<0.001	0.067
**Specificity**	0.008 (0.005–0.010)	<0.001	0.009 (0.007–0.012)	<0.001	0.410
Number of observations:	8,608		8,736		
Pseudo R^2:	0.3806		0.4106		
Overall p value for difference:	0.460				

Younger or older than the median of the study population (45 years).

B. Sex

	Males (n=520)		Females (n=564)		
Attribute	Coefficient (95% CI)	P value for coefficient	Coefficient (95% CI)	P value for coefficient	P value for difference
**Risk factors in model**					
Age	Ref		Ref		
Age and sex	0.241 (0.087–0.395)	0.002	0.236 (0.096–0.375)	0.001	0.955
Age, sex and lifestyle risk score	-0.124 (-0.298–0.050)	0.163	0.009 (-0.150–0.167)	0.914	0.278
Age and genetic risk score	0.217 (0.083–0.351)	0.002	0.261 (0.140–0.382)	<0.001	0.637
**Sensitivity**	0.067 (0.063–0.071)	<0.001	0.061 (0.057–0.064)	<0.001	**0.025**
**Specificity**	0.009 (0.007–0.011)	<0.001	0.008 (0.006–0.010)	<0.001	0.513
Number of observations:	8,320		9,024		
Pseudo R^2:	0.4169		0.3765		
Overall p value for difference:	0.258				

C. Ethnicity

	White (n=934)		Other (n=150)		
Attribute	Coefficient (95% CI)	P value for coefficient	Coefficient (95% CI)	P value for coefficient	P value for difference
**Risk factors in model**					
Age	Ref		Ref		
Age and sex	0.291 (0.178–0.404)	<0.001	-0.032 (-0.299–0.234)	0.813	**0.026**
Age, sex and lifestyle risk score	-0.006 (-0.134–0.123)	0.931	-0.258 (-0.549–0.032)	0.082	0.115
Age and genetic risk score	0.253 (0.154–0.351)	<0.001	0.209 (-0.015–0.433)	0.068	0.729
**Sensitivity**	0.064 (0.062–0.067)	<0.001	0.059 (0.052–0.065)	<0.001	0.129
**Specificity**	0.009 (0.008–0.011)	<0.001	0.004 (0.000–0.009)	0.034	**0.035**
Number of observations:	14,944		2,400		
Pseudo R^2:	0.4034		0.3535		
Overall p value for difference:	**0.011**				

D. Education

	No university education (n=522)		University education (n=561)		
Attribute	Coefficient (95% CI)	P value for coefficient	Coefficient (95% CI)	P value for coefficient	P value for difference
**Risk factors in model**					
Age	Ref		Ref		
Age and sex	0.390 (0.241–0.539)	<0.001	0.097 (-0.048–0.241)	0.189	**0.005**
Age, sex and lifestyle risk score	0.075 (-0.097–0.246)	0.394	-0.164 (-0.325–-0.003)	0.046	0.053
Age and genetic risk score	0.319 (0.190–0.447)	<0.001	0.168 (0.041–0.294)	0.009	0.107
**Sensitivity**	0.063 (0.060–0.067)	<0.001	0.064 (0.060–0.067)	<0.001	0.799
**Specificity**	0.007 (0.004–0.009)	<0.001	0.010 (0.008–0.012)	<0.001	0.061
Number of observations:	8,352		8,976		
Pseudo R^2:	0.4001		0.3933		
Overall p value for difference:	**0.005**				

E. Social grade

	Social grade ABC1 (n=788)		Social grade C2DE (n=287)		
Attribute	Coefficient (95% CI)	P value for coefficient	Coefficient (95% CI)	P value for coefficient	P value for difference
**Risk factors in model**					
Age	Ref		Ref		
Age and sex	0.164 (0.042–0.286)	0.009	0.400 (0.202–0.598)	<0.001	**0.043**
Age, sex and lifestyle risk score	-0.146 (-0.283–-0.008)	0.038	0.180 (-0.049–0.410)	0.124	**0.021**
Age and genetic risk score	0.178 (0.071–0.284)	0.001	0.388 (0.217–0.559)	<0.001	**0.043**
**Sensitivity**	0.064 (0.062–0.067)	<0.001	0.061 (0.057–0.066)	<0.001	0.333
**Specificity**	0.010 (0.008–0.011)	<0.001	0.005 (0.002–0.008)	0.002	**0.019**
Number of observations:	12,608		4,592		
Pseudo R^2:	0.3995		0.3893		
Overall p value for difference:	**0.007**				

Excluding unclear.
Based on the chief income earner's occupation.

F. Smoking status

	Never smokers (n=625)		Current or previous smokers (n=455)		
Attribute	Coefficient (95% CI)	P value for coefficient	Coefficient (95% CI)	P value for coefficient	P value for difference
**Risk factors in model**					
Age	Ref		Ref		
Age and sex	0.192 (0.056–0.328)	0.006	0.304 (0.144–0.464)	<0.001	0.284
Age, sex and lifestyle risk score	-0.096 (-0.249–0.058)	0.223	0.009 (-0.174–0.192)	0.926	0.404
Age and genetic risk score	0.200 (0.082–0.319)	<0.001	0.300 (0.161–0.439)	<0.001	0.295
**Sensitivity**	0.063 (0.060–0.066)	<0.001	0.064 (0.060–0.068)	<0.001	0.749
**Specificity**	0.008 (0.006–0.011)	<0.001	0.009 (0.006–0.011)	<0.001	0.898
Number of observations:	10,000		7,280		
Pseudo R^2:	0.3899		0.4047		
Overall p value for difference:	0.848				

Excluding missing.

G. Family history of cancer

	Family history of cancer (n=429)		No family history of cancer (n=637)		
Attribute	Coefficient (95% CI)	P value for coefficient	Coefficient (95% CI)	P value for coefficient	P value for difference
**Risk factors in model**					
Age	Ref		Ref		
Age and sex	0.403 (0.234–0.572)	<0.001	0.136 (0.003–0.269)	0.045	**0.014**
Age, sex and lifestyle risk score	0.110 (-0.086–0.306)	0.273	-0.145 (-0.294–0.004)	0.056	**0.048**
Age and genetic risk score	0.357 (0.210–0.504)	<0.001	0.167 (0.051–0.283)	0.005	**0.049**
**Sensitivity**	0.066 (0.062–0.070)	<0.001	0.062 (0.059–0.065)	<0.001	0.153
**Specificity**	0.009 (0.006–0.011)	<0.001	0.009 (0.007–0.011)	<0.001	0.962
Number of observations:	6,864		10,192		
Pseudo R^2:	0.4219		0.3820		
Overall p value for difference:	**0.026**				

Excluding missing and don't know.

H. Numeracy

	High numeracy (n=519)		Low numeracy (n=565)		
Attribute	Coefficient (95% CI)	P value for coefficient	Coefficient (95% CI)	P value for coefficient	P value for difference
**Risk factors in model**					
Age	Ref		Ref		
Age and sex	0.275 (0.111–0.439)	0.001	0.238 (0.103–0.372)	0.001	0.725
Age, sex and lifestyle risk score	-0.231 (-0.415–-0.047)	0.014	0.094 (-0.060–0.248)	0.234	**0.009**
Age and genetic risk score	0.206 (0.062–0.350)	0.005	0.291 (0.174–0.408)	<0.001	0.377
**Sensitivity**	0.074 (0.070–0.078)	<0.001	0.056 (0.053–0.059)	<0.001	**<0.001**
**Specificity**	0.016 (0.013–0.018)	<0.001	0.003 (0.001–0.005)	0.003	**<0.001**
Number of observations:	8,304		9,040		
Pseudo R^2:	0.4693		0.3428		
Overall p value for difference:	**<0.001**				

All three numeracy questions correct or not.

I. DCE understanding

Attribute	Good understanding – 2/3 (n=914) Coefficient (95% CI)	P value for coefficient	Poor understanding – 0/1 (n=170) Coefficient (95% CI)	P value for coefficient	P value for difference
**Risk factors in model**					
Age	Ref		Ref		
Age and sex	0.241 (0.125–0.357)	<0.001	0.257 (0.025–0.489)	0.030	0.900
Age, sex and lifestyle risk score	-0.080 (-0.211–0.051)	0.233	0.082 (-0.184–0.347)	0.546	0.305
Age and genetic risk score	0.234 (0.133–0.335)	<0.001	0.301 (0.099–0.503)	0.004	0.566
**Sensitivity**	0.067 (0.064–0.070)	<0.001	0.050 (0.044–0.055)	<0.001	**<0.001**
**Specificity**	0.010 (0.008–0.012)	<0.001	0.001 (-0.003–0.005)	0.671	**<0.001**
Number of observations:	14,624		2,720		
Pseudo R^2:	0.4194		0.2911		
Overall p value for difference:	**<0.001**				

Number of correct DCE understanding questions (out of 3).

J. Attitudes towards cancer screening

Attribute	Yes, for everyone (n=632) Coefficient (95% CI)	P value for coefficient	It depends (n=426) Coefficient (95% CI)	P value for coefficient	P value for difference
**Risk factors in model**					
Age	Ref		Ref		
Age and sex	0.225 (0.087–0.363)	0.001	0.229 (0.066–0.392)	0.006	0.972
Age, sex and lifestyle risk score	-0.092 (-0.248–0.064)	0.246	-0.024 (-0.209–0.161)	0.798	0.586
Age and genetic risk score	0.253 (0.134–0.372)	<0.001	0.199 (0.055–0.342)	0.007	0.577
**Sensitivity**	0.065 (0.062–0.069)	<0.001	0.063 (0.059–0.066)	<0.001	0.369
**Specificity**	0.006 (0.004–0.008)	<0.001	0.012 (0.010–0.015)	<0.001	**0.001**
Number of observations:	10,112		6,816		
Pseudo R^2:	0.4046		0.3954		
Overall p value for difference:	**0.003**				

"Do you think that benefits of cancer screening outweigh the possible side effects, potential harms and inconvenience?" Yes for everyone versus it depends on your age and sex, the type of cancer, how you feel about cancer and/or how you feel about screening tests (no for everyone excluded, n=24).

K. Ease of completing the DCE

Attribute	Easy (n=564) Coefficient (95% CI)	P value for coefficient	Difficult (n=520) Coefficient (95% CI)	P value for coefficient	P value for difference
**Risk factors in model**					
Age	Ref		Ref		
Age and sex	0.294 (0.132–0.455)	<0.001	0.189 (0.053–0.325)	0.007	0.323
Age, sex and lifestyle risk score	-0.212 (-0.393–-0.031)	0.022	0.051 (-0.103–0.206)	0.514	0.033
Age and genetic risk score	0.291 (0.153–0.430)	<0.001	0.206 (0.085–0.326)	0.001	0.373
**Sensitivity**	0.077 (0.073–0.081)	<0.001	0.053 (0.050–0.056)	<0.001	**<0.001**
**Specificity**	0.010 (0.007–0.012)	<0.001	0.007 (0.005–0.009)	<0.001	0.145
Number of observations:	9,024		8,320		
Pseudo R^2:	0.4873		0.3129		
Overall p value for difference:	**<0.001**				

CI: confidence interval. Ref: reference.

Supplementary Table 6: Sensitivity analysis – Conditional logit regression using underlying attributes plus relative importance of attributes comparing participants who passed and failed the rationality check.

	**Passed rationality check (n=1,084)**		**All respondents (n=1,172)**		
Attribute	Coefficient (95% CI)	P value for coefficient	Coefficient (95% CI)	P value for coefficient	P value for difference
**Risk factors in model**					
Age	Ref		Ref		
Age and sex	0.239 (0.136–0.342)	<0.001	0.226 (0.135–0.317)	<0.001	0.584
Age, sex and lifestyle risk score	-0.051 (-0.168–0.066)	0.394	0.017 (-0.087–0.121)	0.754	**0.018**
Age and genetic risk score	0.242 (0.152–0.332)	<0.001	0.248 (0.167–0.329)	<0.001	0.763
**Sensitivity**	0.063 (0.061–0.066)	<0.001	0.054 (0.052–0.056)	<0.001	**<0.001**
**Specificity**	0.008 (0.007–0.010)	<0.001	0.007 (0.006–0.009)	<0.001	**0.008**
Number of observations:	17,344		18,752		
Pseudo R^2:	0.3953		0.3258		
Overall p value for difference:	**<0.001**				

CI: confidence interval. Ref: reference.

Supplementary Table 7: Frequency and illustrative quotations of respondents' approaches to selecting between programmes in the DCE.

Thematic analysis of free-text comments given in response to the question, "Please tell us why you found choosing between the different programmes easy or difficult", grouped by whether respondents found choosing between the programmes easy or difficult.

Approach	Easy (n=555)	Difficult (n=544)
**Weighed/balanced the attributes**	• N=78<br>• "There is a balance to be drawn between false positives and missed cancers. This is the key issue when deciding." [69 years, male, White ethnicity, no cancer history]	• N=208<br>• "You had to weigh up which factors were more important than other despite all of them being quite important to the decision." [19 years, male, Black ethnicity, no cancer history]<br>• "The variables changed for each scenario – balancing the respective variables is therefore difficult. In one programme what might seem a reasonable number for one variable might be compromised by a less reasonable number for another factor, yet in the other programme the weight of those ratios might be opposite. There sometimes isn't a 'right' or easy answer." [53 years, male, White ethnicity, no cancer history]
**Prioritised certain attributes**	• N=400<br>• "I had a clear sense of my hierarchy of importance in terms of the different factors, so it was always relatively clear which programme to choose." [20 years, female, White ethnicity, no cancer history]	• N=119<br>• "There are so many factors to consider and I found myself prioritising those factors and what that meant for the patient." [47 years, female, Black ethnicity, no cancer history]

Out of a total 1,148 free-text comments, 1,099 comments indicated respondents' approach for selecting between programmes**.**

Supplementary Figure 1: Ease of completing the DCE by study arm.

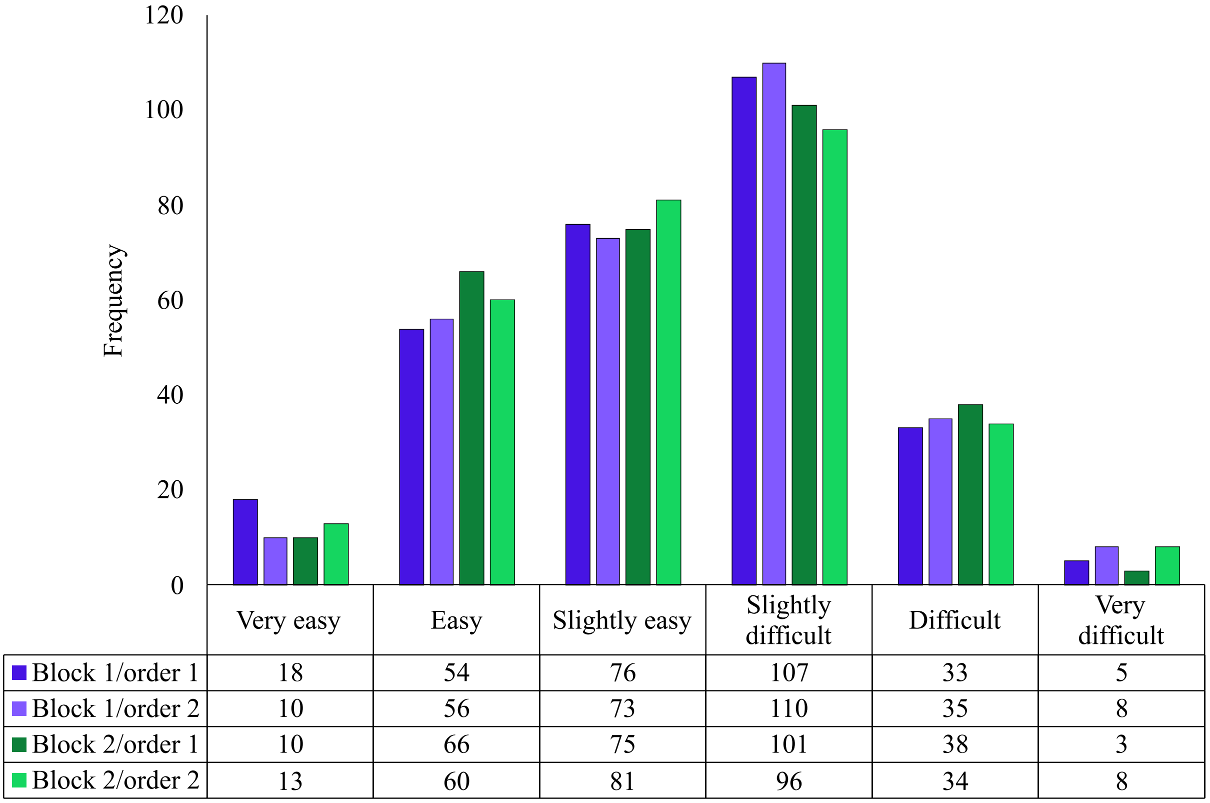

	Very easy	Easy	Slightly easy	Slightly difficult	Difficult	Very difficult
Block 1/order 1	18	54	76	107	33	5
Block 1/order 2	10	56	73	110	35	8
Block 2/order 1	10	66	75	101	38	3
Block 2/order 2	13	60	81	96	34	8

N respondents.

Supplementary Figure 2: Participants' ranking of attributes included in the DCE.

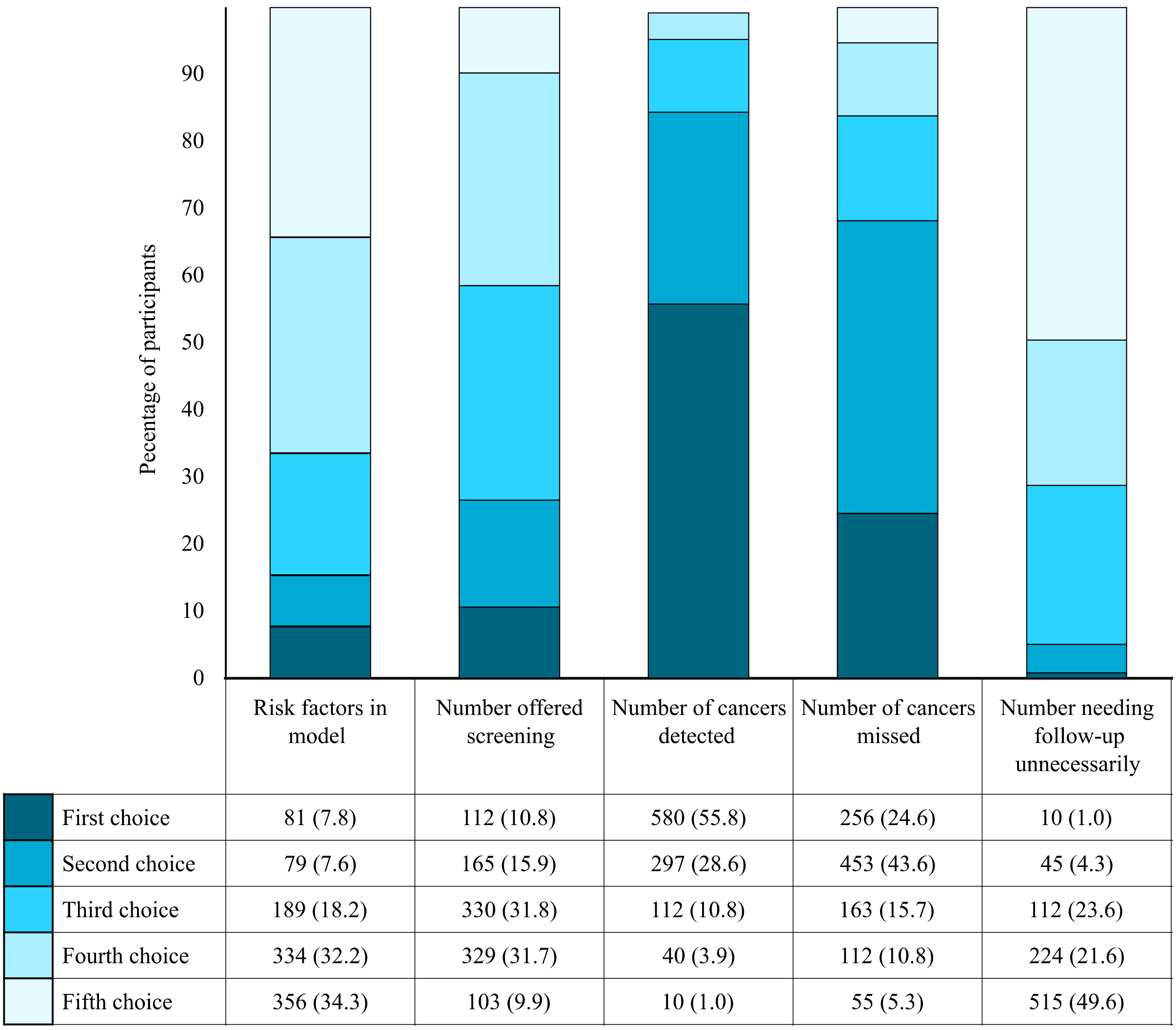

	Risk factors in model	Number offered screening	Number of cancers detected	Number of cancers missed	Number needing follow-up unnecessarily
First choice	81 (7.8)	112 (10.8)	580 (55.8)	256 (24.6)	10 (1.0)
Second choice	79 (7.6)	165 (15.9)	297 (28.6)	453 (43.6)	45 (4.3)
Third choice	189 (18.2)	330 (31.8)	112 (10.8)	163 (15.7)	112 (23.6)
Fourth choice	334 (32.2)	329 (31.7)	40 (3.9)	112 (10.8)	224 (21.6)
Fifth choice	356 (34.3)	103 (9.9)	10 (1.0)	55 (5.3)	515 (49.6)

N (%). n=1,039 (respondents with valid ranking of attributes and who passed the rationality check).
